# Effects of cardiac rehabilitation on left ventricle function and mass evaluated by cardiac magnetic resonance imaging in post myocardial infarction patients

**DOI:** 10.1186/1532-429X-13-S1-P167

**Published:** 2011-02-02

**Authors:** Nataly L Izeli, Julio C Crescencio, Antonio Pazin-Filho, Lourenco Gallo, Andre Schmidt

**Affiliations:** 1 Medical School of Ribeirao Preto-University of Sao Paulo, Ribeirao Preto, SP, Brazil

## Background

Numerous studies show the benefits of cardiac rehabilitation in patients with coronary artery disease. Nevertheless, the effects of regular exercise on myocardial function are still controversial.

## Objective

This study aimed to evaluate the effects of aerobic exercise of moderate intensity, performed in patients after a first myocardial infarction on cardiac function by cardiac magnetic resonance.

## Methods

Twenty-six male patients, 52.9 ± 7.9 years, after a first myocardial infarction were assigned in two groups: 18 in trained group (TG) and eight in control group (CG). The TG performed supervised aerobic exercise on treadmill at 50 to 70% of heart rate reserve, twice a week. The groups were followed for at least three months. Laboratory tests, anthropometric measurements, exercise test and cardiac magnetic resonance were conducted before and after intervention. Statistical analysis was performed using the nonparametric tests with a significance level of 5%.

## Results

After intervention, there was a statistically significant reduction of 10.8% in fasting blood glucose in TG (p = 0.011). There was a statistically significant increase in weight and body mass index of the CG (p = 0.0391). There was a statistically significant increase in oxygen consumption obtained indirectly in the treadmill exercise test only in the TG (35.4 ± 8.1 to 49.1 ± 9.6 ml/kg/min, p < 0.0001). The chronotropic index had a statistically significant increase (p = 0.0432) of 12.65% in GT and did not change in CG. The Duke score showed statistically significant difference (p = 0.001) in values obtained in the TG, with the initial score of 6.9 ± 5.2 and final of 10.9 ± 4.1. These same trends were not found in the CG. Cardiac magnetic resonance showed a statistically significant reduction in ventricular mass from 128.7 ± 38.9 to 117.2 ± 27.2 g in TG (p = 0.0032). There was a trend to increase of left ventricle end diastolic volume (LVEDV) in both groups. No significant change occurred in left ventricle ejection fraction in both groups. The relation LV mass/LVEDV reduced from 1.29±0.36 to 1.05±0.22 g/ml.

## Conclusions

It was observed that aerobic exercise of moderate intensity improved physical capacity and other cardiovascular variables, and identified a trend to a positive remodeling in the trained group, where the increase in left ventricular diastolic dimension was not associated with increased in ventricular mass.

